# HDAC-Specific Inhibitors Induce the Release of Porcine Epidemic Diarrhea Virus via the COPII-Coated Vesicles

**DOI:** 10.3390/v15091874

**Published:** 2023-09-04

**Authors:** Ying Yang, Huan Chen, Caisheng Zhang, Hyun-Jin Shin, Yingjuan Qian, Yong-Sam Jung

**Affiliations:** 1 One Health Laboratory, Jiangsu Foreign Expert Workstation, College of Veterinary Medicine, Nanjing Agricultural University, Nanjing 210095, China; 2018207012@njau.edu.cn (Y.Y.); chenhuan2019@njau.edu.cn (H.C.); 2020107034@stu.njau.edu.cn (C.Z.); 2 MOE Joint International Research Laboratory of Animal Health and Food Safety, College of Veterinary Medicine, Nanjing Agricultural University, Nanjing 210095, China; 3 College of Veterinary Medicine, Chungnam National University, Daejeon 34134, Republic of Korea; shin0089@cnu.ac.kr; 4 Jiangsu Key Laboratory for High-Tech Research and Development of Veterinary Biopharmaceuticals, Jiangsu Agri-Animal Husbandry Vocational College, Veterinary Bio-Pharmaceutical, Taizhou 225300, China

**Keywords:** HDAC-specific inhibitors, COPII-coated vesicles, porcine epidemic diarrhea virus, viral release

## Abstract

Porcine epidemic diarrhea virus (PEDV) is an alpha-coronavirus causing acute diarrhea and high mortality in neonatal suckling piglets, resulting in huge economic losses for the global swine industry. The replication, assembly and cell egression of PEDV, an enveloped RNA virus, are mediated via altered intracellular trafficking. The underlying mechanisms of PEDV secretion are poorly understood. In this study, we found that the histone deacetylase (HDAC)-specific inhibitors, trichostatin A (TSA) and sodium butyrate (NaB), facilitate the secretion of infectious PEDV particles without interfering with its assembly. We found that PEDV N protein and its replicative intermediate dsRNA colocalize with coat protein complex II (COPII)-coated vesicles. We also showed that the colocalization of PEDV and COPII is enhanced by the HDAC-specific inhibitors. In addition, ultrastructural analysis revealed that the HDAC-specific inhibitors promote COPII-coated vesicles carrying PEDV virions and the secretion of COPII-coated vesicles. Consistently, HDAC-specific inhibitors-induced PEDV particle secretion was abolished by Sec24B knockdown, implying that the HDAC-specific inhibitors-mediated COPII-coated vesicles are required for PEDV secretion. Taken together, our findings provide initial evidence suggesting that PEDV virions can assemble in the endoplasmic reticulum (ER) and bud off from the ER in the COPII-coated vesicles. HDAC-specific inhibitors promote PEDV release by hijacking the COPII-coated vesicles.

## 1. Introduction

Coronaviruses (CoVs), belonging to the order *Nidovirales* under the family *Coronaviridae*, are enveloped viruses with a positive-sense single-stranded RNA genome. The Coronaviridae family includes two subfamilies: *Coronavirinae* and *Torovirinae*. The Coronavirinae subfamily is further classified into four genera, the alpha-, beta-, gamma- and delta-coronaviruses, based on genotypic and serological characterizations [1,2,3]. The notorious severe acute respiratory syndrome coronavirus (SARS-CoV) and severe acute respiratory syndrome coronavirus 2 (SARS-CoV-2) represent beta-coronaviruses [4]. Porcine epidemic diarrhea virus (PEDV), an alpha-coronavirus in the family *Coronaviridae*, causes severe watery diarrhea, vomiting, dehydration and high mortality in neonatal suckling piglets, resulting in huge economic losses in the global swine industry [5,6,7].

CoVs and others enveloped viruses, use and modify intracellular compartments of the secretory pathway to facilitate their replication, assembly and egression by hijacking the host cell´s transport machinery [8]. Viral particles assemble in the ER-Golgi intermediate compartment (ERGIC)/Golgi compartment, and is a general hallmark of CoVs [8,9]. Previous reports revealed that alpha-coronaviruses (TGEV, HCoV-NL36) and beta-coronaviruses (MHV) assemble in the ER during the late stages of virus infection. Other studies have indicated that PEDV particles assemble in both the ER and Golgi apparatus [8,10,11,12,13]. The egress pathway of PEDV is triggered by the interaction between the nucleocapsid (N) protein loaded with the newly synthesized genomic RNA and the structural spike (S), envelope (E), and membrane (M) proteins, budding into the lumen of the ER and the ERGIC. Virus particles reach the Golgi and trans-Golgi network (TGN) by vesicular transport for glycosylation and other post-translational modifications. Mature virions are subsequently released via fusion of smooth-walled vesicles with the plasma membrane similar to exocytosis [13,14]. However, the underlying mechanisms of PEDV secretion have yet to be fully understood.

In eukaryotic cells, the conventional secretory transport pathway is characterized by the sequential transport of newly synthesized lipids and proteins from the ER to the plasma membrane by transport vesicles via the ERGIC and the Golgi apparatus [15,16]. Intracellular trafficking vesicles are mainly composed of COPI-, COPII- and clathrincoated vesicles (CCVs) [17,18]. COPII-coated vesicles mediate the transport of cargo from the ER to the Golgi apparatus [19]. COPI-coated vesicles mediate cargo transport from the Golgi apparatus to the ER, or between Golgi cisternae [20,21]. CCVs mediate cargo transport from the TGN to the endosomes and the endocytosis of cargo at the plasma membrane [17,22]. The COPII-coated machinery is composed of five cytosolic proteins: Sar1, Sec23, Sec24, Sec13 and Sec31 [23]. The assembly of COPII-coated vesicle is initiated by the recruitment and activation of the small cytoplasmic GTPase Sar1 by the GTP exchange factor (GEF) Sec12. Sar1-GTP inserts into the ER membrane and recruits the Sec23/Sec24 heterodimer by binding to the Sec23. Sec23 acts as a GTPase activating protein (GAP) for Sar1 and Sec24 participates in cargo selection. Sar1 carrying a cargo-loaded Sec23/Sec24 heterodimer forms a so-called “pre-budding complex” and in turn recruits the Sec13/Sec31 heterotetramer onto the “pre-budding complex” to complete vesicle formation [16,23,24,25,26,27]. As the canonical secretory pathway for ER export, COPII-coated vesicles are frequently hijacked for viral genome replication and transport of viral particles. For example, poliovirus uses COPII-coated vesicles for the formation of replication complexes (RCs) [28,29]. Parvovirus particles [30], hepatitis B subviral envelope particles [31], hepatitis C virus (HCV) lipoviroparticles [32], rotavirus NSP4 [33], Ebola and Marburg virus matrix protein VP40 utilize the COPII transport system for intracellular transport [34].

HDAC inhibitors are approved by the United States Food and Drug Administration (FDA) as cancer therapeutics and are candidate therapies for other diseases, including arthritis, cardiac disease, inflammatory diseases and a few neurological disorders [35,36,37]. In addition, HDAC inhibitors have been found to exert antiviral effects. TSA and Suberoylanilide hydroxamic acid (SAHA) suppress respiratory syncytial virus (RSV) and HCV replication [38,39,40]. Romidepsin prevents the entry of SARS-CoV-2 [41]. In addition, class I-selective HDAC inhibitors enhance HIV latency reversal [42].

This study investigated the relationship between HDAC-specific inhibitors and COPII-coated vesicles during PEDV infection. We showed that the HDAC-specific inhibitors, TSA and NaB, facilitate the secretion of infectious PEDV particles. We also found that PEDV particles utilize the COPII-coated vesicles for intracellular transport. In addition, transmission electron microscopy (TEM) and immunoelectron microscopy (IEM) revealed that secretion of COPII-coated vesicles carrying PEDV virions is promoted by HDAC-specific inhibitors treatment. Finally, we also demonstrated that the knockdown of Sec23A or Sec24B suppresses HDAC-specific inhibitors-induced PEDV secretion. Taken together, this study establishes that HDAC-specific inhibitors-induced COPII-coated vesicles are essential for PEDV release.

## 2. Materials and Methods

### 2.1. Cell Culture

African green monkey Cercopithecus aethiops kidney epithelial cells (Vero-E6 cells) were cultured in Dulbecco’s Modified Eagle Medium (DMEM) (Invitrogen, Carlsbad, CA, USA) supplemented with 8% fetal bovine serum (Pan-Biotech, Inc. Aidenbach, Germany) and 1% penicillin and streptomycin (MDBio, Qingdao, China) at 37 °C in a 5% CO_2_ incubator.

### 2.2. Antibodies and Reagents

Rabbit polyclonal anti-Sec21p was supplied by Agrisera (Vännäs, Sweden). Rabbit polyclonal anti-COPII was purchased from Invitrogen (Carlsbad, CA, USA). Rabbit polyclonal anti-clathrin was provided by Abcam (Cambridge, UK). Rabbit monoclonal an-ti-Sec24B (D7D6S) was obtained from Cell Signaling Technology, Inc. (Danvers, MA, USA). Rabbit polyclonal anti-Ac-Histone H3 (Lys 9/14) was ordered from Santa Cruz (Dallas, TX, USA). Rabbit polyclonal anti-actin (A2066) antibodies were purchased from Merck, Inc. (Darmstadt, Germany). Mouse polyclonal anti-PEDV-N and rabbit polyclonal anti-PEDV-N antibodies were previously generated in the lab [3]. The horseradish peroxidase (HRP)-conjugated goat anti-rabbit IgG and HRP-conjugated goat anti-mouse IgG antibodies were ordered from MiliporeSigma (Merck Inc., Darmstadt, Germany). Alexa 488-conjugated goat anti-rabbit IgG (A-11001) and Alexa 555-conjugated goat anti-mouse IgG (A-21428) antibodies were supplied by Thermo Fisher Scientific, Inc. (Waltham, MA, USA). The 10 nm labeled goat anti-rabbit secondary antibody (G7402) was purchased from Sigma-Aldrich (St. Louis, MO, USA). TSA, and NaB were provided by Selleck (Houston, TX, USA). Glutaraldehyde (2.5%, pH 7.4) and paraformaldehyde (4%)-glutaraldehyde (0.5%) mixture, (pH 7.4) were purchased from Yuanye Bio-Technology Co., Ltd., (Shanghai, China). Ethanol was obtained from Sinaopharm Group Chemical Reagent Co. LTD (Shanghai, China). LR White Resin was supplied by HaideBio (Beijing, China). Enhanced chemiluminescence (ECL) reagent was purchased from Youqing Biology (Nanjing, China).

### 2.3. Viral Infection and Titer Determination 

PEDV (strain HLJBY) was cultured in Vero-E6 cells. The cells were infected with PEDV in DMEM without FBS and incubated at 37 °C for 1 h. After incubation, the cells were washed with phosphate-buffered saline (PBS) and transferred to DMEM containing 2% FBS and 17.5 ng of trypsin per mL. The virus titer was determined using the plaque formation assay. Briefly, 6-well plates were seeded with 1 × 10^6^ Vero-E6 cells/well the day before inoculation. The cells were washed with PBS, and 10-fold serial dilutions (10^2^ to 10^7^) of viruses were incubated with a confluent monolayer of Vero-E6 cells at 37 °C for 1 h. After incubation, the cells were washed with PBS, followed by the addition of 2 mL overlay medium (2% low-melting-point agarose (Lonza Inc., Basel, Switzerland) in 2 × DMEM with 2% FBS and 17.5 ng of trypsin per mL). The plates were incubated at 37 °C with 5% CO_2_ for 2 to 3 days. The cells were stained with 0.5% crystal violet.

### 2.4. Western Blotting Analysis

Whole-cell extracts were prepared with 2 × SDS sample buffer (4% SDS, 0.1 M Tris HCI pH 6.8, 20% glycerol, 2% bromophenol blue, and 10% β-mercaptoethanol) and boiled for 10 min at 98 °C. Next, the samples were subjected to sodium dodecyl sulfate-polyacrylamide gel electrophoresis (SDS-PAGE) and then transferred to a nitro-cellulose transfer membrane (Pall Corporation, Port Washington, NY, USA) using a Mighty Small Trans-fer Tank system (Hoefer, MA, USA). Then, the membranes were blocked with 3% non-fat milk in PBS with 0.5% Tween 20 (PBST) for 30 min at room temperature and then incubated with a specific primary antibody overnight at 4 °C followed by incubation with secondary antibodies for 4 h at 4 °C. The positive bands were visualized with the enhanced chemiluminescence (ECL) reagent, and imaged using a BioSpectrum Imaging System (UVP, Upland, CA, USA).

### 2.5. Knockdown of Sec23A/Sec24B Expression

The siRNA oligonucleotides against Sec24B and a nontargeting control siRNA were purchased from Biotend, Inc. (Shanghai, China). For siRNA gene knockdown experiments, 3.6 × 10^5^ Vero-E6 cells were seeded into 6-well plates/well for 18 h and transfected with 50 nM siRNA oligonucleotide using 7.5 μL Mirus following the manufacturer’s instructions (Madison, WI, USA). After 48 h of transfection, the cells were analyzed by immunoblotting to determine the knockdown efficiency. The Sec23A and Sec24B RNA interference (RNAi) target sequences were as follows: siSec23A-1, 5′-AAG GAA UCA GUU UCC ACC UAG UUA U-3′, siSec23A-2,5′-CCU ACA GCU UUG GUU GGA CUU AUU A-3′; siSec24B-1,5′-CGG UAU AUU CUG GAU UCC AAC AGU A-3′, siSec24B-2,5′-CCC GAU CUU AUG GAG AGC CUC AUA A-3′.

### 2.6. Immunofluorescence Microscopy

The Vero-E6 cells were grown on coverslips and pretreated with or without TSA for 2 h, and then infected with PEDV for different durations. The cells on the coverslips were fixed and permeabilized with 4% formaldehyde and 0.1% Triton X-100 at 37 °C for 30 min. After washing with glycine-PBS, the cells were blocked with 3% BSA in PBS at 37 °C for 30 min. The coverslips were incubated with primary antibody (1:200) at 37 °C for 1 h, followed by secondary antibody (1:500) at 37 °C for 30 min. Unbound antibodies were removed by washing with PBST three times. The nuclei were stained with 4′,6-diamidino-2-phenylindole (DAPI) containing the anti-fade Dabco solution (Thermo Fisher Scientific, Waltham, MA, USA). Images of Figures 3 and 4A were obtained with a confocal microscopy (Nikon Eclipse Ti, A1, Tokyo, Japan) and Figure 6C,D and Figure S2 were obtained using a fluorescence microscopy (Nikon Eclipse Ti-U, Tokyo, Japan). 

### 2.7. Transmission Electron Microscopy/Immunoelectron Microscopy 

Vero-E6 cells were pretreated with or without TSA and NaB for 2 h and then mock infected or infected with PEDV at a MOI = 0.1. After 12 or 24 h, cells were collected and centrifuged into a cell mass. First, the cell mass was fixed in cold glutaraldehyde (2.5%, pH 7.4, TEM) or cold paraformaldehyde (4%)-glutaraldehyde (0.5%) glutaraldehyde mixture, (pH 7.4, IEM) at 4 °C overnight, followed by additional steps including cleaning, fixation, dehydration, resin penetration, embedding, solidification, polymerization, microtomy, and staining. The precipitation was washed three times in 0.1 M PB (pH 7.4), for 10 min each time. It was fixed with 1% osmic acid and then cleaned. The fixed cell mass was gradient dehydrated in different concentrations of ethanol. Resin penetration with different concentrations of embedding agent was performed. An embedding capsule was used for embedding, at 4 °C. The solidification step was performed in a 55°C incubator for 48 h for TEM. For more than 48 h, polymerization was conducted at −20 °C with low temperature UV polymerizer-UVCC2515 (Zhongjingkeyi Technology, Beijing, China) for IEM. Ultrathin sections (70–80 nm) from the resin blocks were obtained using a Leica UC7-ultramicrotome (Wetzlar, Germany), and the tissues were fished out onto 150-meshes nickel grids (Zhongjingkeyi Technology, Beijing, China) with Formvar film; the sections were stored at 4 °C. Immunolabelling was performed by incubating with an anti-COPII antibody overnight at 4 °C, followed by incubation with 10 nm labeled goat anti-rabbit secondary antibody (20 min at room temperature; 1 h at 37 °C; 30 min at room temperature) for IEM. For TEM/IEM, sample was stained with 2% uranyl acetate and lead citrate. Sections were examined with a Hitachi H-7650 (Hitachi, Tokyo, Japan) TEM at an accelerating voltage of 80 kV.

### 2.8. Cell Viability Analysis 

The cytotoxicity of HDAC-specific inhibitors was measured via a Cell Counting Kit-8 (CCK-8) assay, following the manufacturer’s instructions (APExBIO Technology, Inc., Houston, TX, USA). In brief, Vero-E6 cells were seeded in the 96-well plate and cultured for 24 h and then treated with an increased dose of HDAC inhibitors for 12 or 24 h, followed by incubation with CCK-8 reagent (10 μL/well) at 37 °C for 4 h. Finally, the absorbance at 450 nm was measured in an enzyme-linked immunosorbent assay reader. 

### 2.9. Statistical Analysis

The results shown are representative of three replicate experiments. All statistical tests were conducted using GraphPad Prism 7.0 software (San Diego, CA, USA) and are presented as means plus or minus standard deviation (SDs). Statistical significance was determined using the Student’s *t* test. *p* values of <0.05 were considered statistically significant.

## 3. Results

### 3.1. HDAC-Specific Inhibitors Facilitate PEDV Particle Secretion

**Figure 1:**
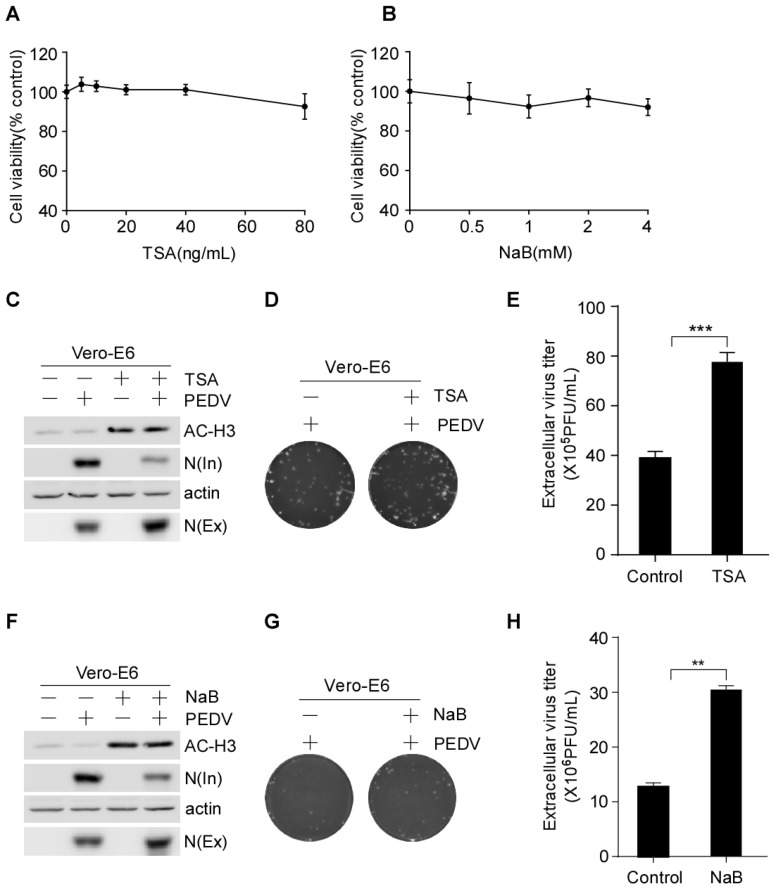
HDAC-specific inhibitors promote the secretion of PEDV virus particles into the extracellular compartment. (**A**,**B**) Determination of the cell viability following treatment with TSA and NaB via CCK-8 test. Vero-E6 cells were pretreated with or without TSA (40 ng/mL) and NaB (2 mM) for 2 h, and then mock-infected or infected with HLJBY (MOI = 0.1). After HLJBY adsorption for 1 h. The cells were further cultured in fresh medium in the presence of TSA and NaB at 12 or 24 h. The infected cell lysates were prepared, and AC-H3, intracellular N, and actin were detected by Western-blot (**C**,**F**). The culture medium was divided into two parts; one part was analyzed by Western blotting of extracellular N (**C**,**F**), and the other part was titrated by plaque formation assay to measure the extracellular PEDV virus titers (**D**,**G**). Graphs show changes in virus titers (**E**,**H**); 12 h (×10^5^), 24 h (×10^6^). Student’s *t* test was used for statistical analysis. **, *p* < 0.01; ***, *p* < 0.001. The error bars indicate standard deviation from three independent experiments. In; intracellular, Ex; extracellular.

HDAC inhibitors, such as TSA and SAHA, suppress HCV and RSV replication [38,39,40], whereas romidepsin inhibits the entry of SARS-CoV-2 [41]. This finding prompted us to investigate whether HDAC inhibitors regulate PEDV infection. To verify this possibility, Vero-E6 cells were pretreated with or without TSA and NaB, inhibitors of class I and II HDACs [43,44], and then infected with PEDV. The culture medium and the infected cells were harvested separately. To test the cytotoxicity of the HDAC-specific inhibitors we performed CCK-8 assay. We found that the HDAC-specific inhibitors at concentrations used in this study did not affect the cell viability (Figure 1A,B). To determine the functionality of HDAC-specific inhibitors, the infected cell lysates were analyzed via Western blotting analysis of acetylated histone H3. The protein level of acetylated histone H3 was increased by the HDAC-specific inhibitors treatment (Figure 1C,F). The infected cell lysates and the culture medium were subjected to Western blotting to analyze the intracellular and extracellular PEDV N protein levels. We found that the HDAC-specific inhibitors decreased the intracellular PEDV-N protein levels but increased the extracellular PEDV-N protein levels (Figure 1C,F and Figure S1). Cells treated with TSA secreted about 2.0-fold higher levels of infectious virus particles in the supernatant than the DMSO-treated cells (Figure 1D,E). Cells treated with NaB secreted infectious virus particles in the supernatant about 2.3-fold higher than the control cells (Figure 1G,H). These data suggested that the HDAC-specific inhibitors facilitate the secretion of PEDV virus particles into the extracellular medium. 

**Figure 2:**
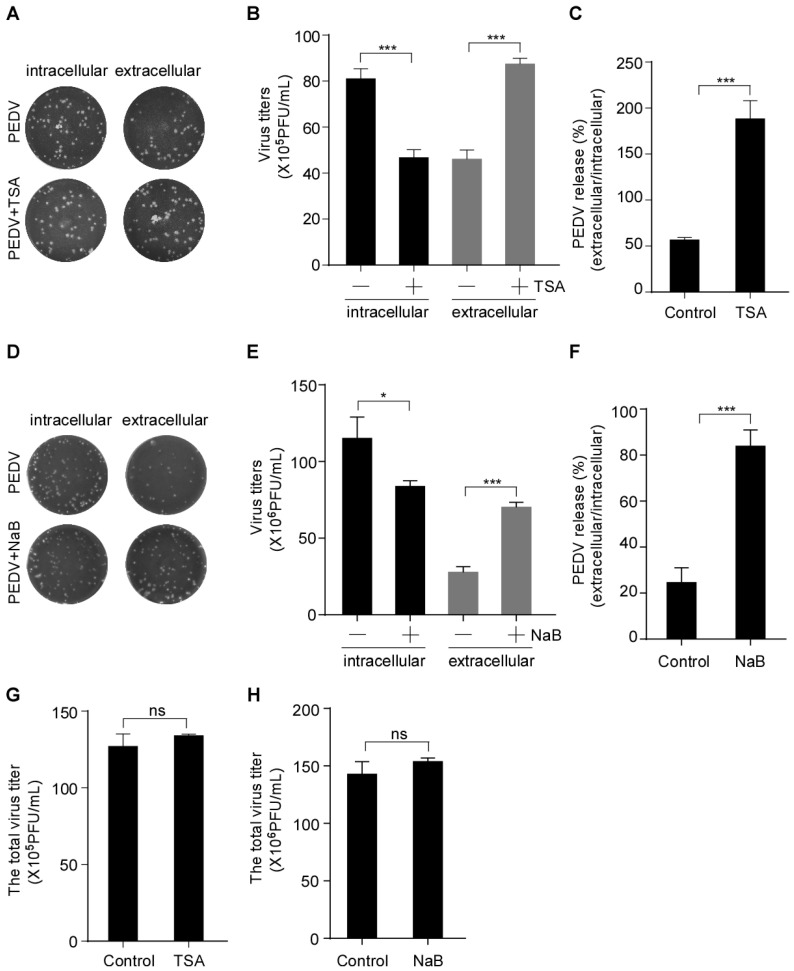
HDAC-specific inhibitors facilitate the secretion of infectious PEDV particles. Vero-E6 cells were pretreated with or without TSA (40 ng/mL) and NaB (2 mM) for 2 h, and then mock-infected or infected with HLJBY (MOI = 0.1). After HLJBY adsorption for 1 h, the cells were further cultured in fresh medium in the presence of TSA and NaB at 12 or 24 h. The culture medium was first collected, and the infected cells were washed thrice with phosphate-buffered saline (PBS) before adding fresh medium, and then lysed via three freeze/thaw cycles to obtain the intracellular PEDV. (**A**,**D**) The virus titers (intracellular and extracellular) were detected by plaque formation assay. (**B**,**E**) Graphs show changes in virus titers (intracellular and extracellular); 12 h (×10^5^), 24 h (×10^6^). (**C**,**F**) Graphs show PEDV release (ratio of extracellular to intracellular virus titers). (**G**,**H**) Graphs show changes in total virus titer. Student’s *t* test was used for statistical analysis. The error bars indicate standard deviation from three independent experiments. ns, *p* > 0.05; *, *p* < 0.05; ***, *p* < 0.001.

To further analyze the effect of HDAC-specific inhibitors on PEDV infection, Vero-E6 cells were pretreated with or without TSA and NaB and then infected with PEDV. The intracellular and extracellular virus titers were determined by plaque formation assay (PFU). PFU results showed that the HDAC-specific inhibitors treatment decreased the intracellular virus titer and increased the extracellular virus titer (Figure 2A,B,D,E). We used the ratio of virus titers in extracellular and intracellular compartments to evaluate virus release. The results showed that the TSA treatment resulted in an approximately 3.3-fold increase in the release of infectious virus particles (Figure 2C) compared with the approximately 2.7-fold increased by NaB treatment (Figure 2F). However, the total virus titer of HDAC-specific inhibitors-treated cells did not change significantly compared with the control (Figure 2G,H). These data indicated that the HDAC-specific inhibitors promote PEDV release without affecting its assembly.

### 3.2. Colocalization of PEDV N Proteins and Its Replication Complex with COPII-Coated Vesicles

**Figure 3:**
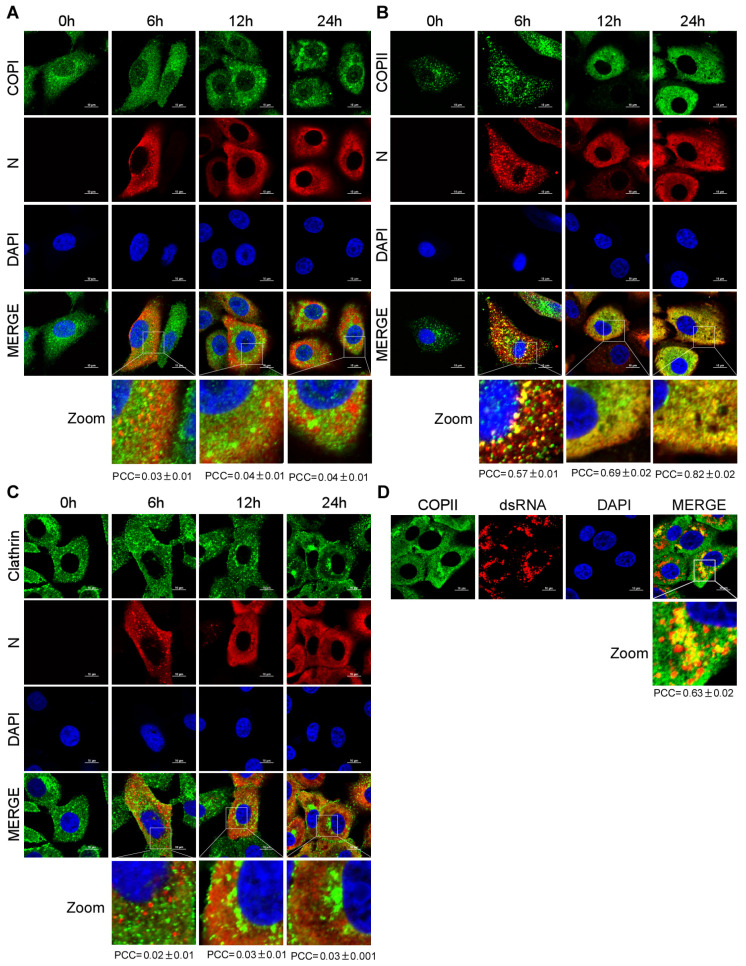
PEDV N protein and its replicative intermediate dsRNA colocalize with COPII-coated vesicles. Vero-E6 cells infected with HLJBY (MOI = 0.1) for 0, 6, 12 and 24 h. The cells were fixed and stained with Sec21p (γ subunit of COPI) antibody (**A**); COPII antibody (**B**); clathrin antibody (**C**); and Alexa 488-conjugated goat anti-rabbit IgG antibodies (green) and then stained with PEDV-N antibody and Alexa 555-conjugated goat anti-mouse IgG antibody (red). The nuclei were stained with DAPI (blue). Images were acquired with a Nikon confocal microscopy. Bottom panel shows a magnified view of the boxed area in panels (**A**–**C**) (Merged). For quantitative colocalization analysis (QCA), Pearson correlation coefficient (PCC) values were calculated and represent the mean ± SD. (**D**) Vero-E6 cells infected with HLJBY (MOI = 0.1) for 24 h. The cells were fixed and stained with COPII antibody and Alexa 488-conjugated goat anti-rabbit IgG anti-bodies (green), and then stained with dsRNA antibody and Alexa 555-conjugated goat anti-mouse IgG antibody (red). The nuclei were stained with DAPI (blue). Images were acquired with a Nikon confocal microscopy. Bottom panel shows a magnified view of the boxed area in panel (**D**) (Merge). For quantitative colocalization analysis (QCA), PCC values were calculated and represent the mean ± SD.

It has been reported that PEDV utilizes smooth-walled vesicles to egress [14]. Previous studies have shown that intracellular trafficking vesicles in eukaryotic cells are mainly composed of COPI, COPII and CCVs [17,18]. To determine the vesicular transport involved in PEDV transport, Vero-E6 cells were infected with PEDV. At different time points, Vero-E6 cells were stained with anti-Sec21p (γ subunit of COPI), anti-COPII and anti-clathrin antibodies to detect transport vesicles. The subcellular localization of PEDV N protein and trafficking vesicle markers was examined using a confocal microscopy. The confocal microscopic images showed that only COPII colocalized with PEDV N protein and the PEDV infection enhanced the colocalization of COPII and PEDV N in a time-dependent manner (Figure 3B). Neither COPI nor clathrin colocalized with PEDV N protein at the indicated time points (Figure 3A,C). Vero-E6 cells were also stained with anti-dsRNA antibody to detect the virus replication complex. We found that COPII colocalized with PEDV replicative intermediate dsRNA (Figure 3D). These findings suggested that COPII-coated vesicles mediate PEDV trafficking.

### 3.3. PEDV N Protein Is Efficiently Captured by The COPII-Coated Vesicles upon HDAC-Specific Inhibitors Treatment

**Figure 4:**
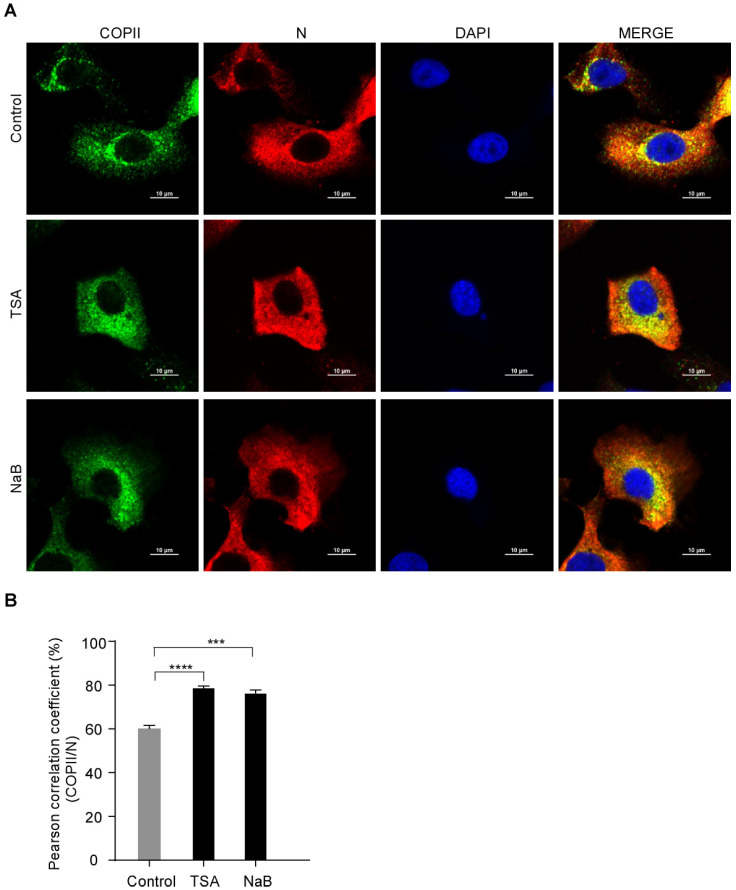
The colocalization between PEDV N and COPII is enhanced by HDAC-specific inhibitors. Vero-E6 cells were pretreated with or without TSA (60 ng/mL) and NaB (4 mM) for 2 h, and then infected with HLJBY (MOI = 1) for 1 h. The cells were further cultured in fresh medium in the presence of TSA and NaB at 8 h. (**A**) The cells were fixed and stained with COPII antibody and Alexa 488-conjugated goat anti-rabbit IgG antibodies (green) and then stained with PEDV-N antibody and Alexa 555-conjugated goat anti-mouse IgG antibody (red). The nuclei were stained with DAPI (blue). The images were acquired with a Nikon confocal microscopy. (**B**) Graphs show the PCC of COPII and PEDV N. For quantitative colocalization analysis (QCA), PCC values were calculated and represent the mean ± SD. Student’s *t* test was used for statistical analysis. ***, *p* < 0.001; ****, *p* < 0.0001.

Our study showed that the HDAC-specific inhibitors facilitated PEDV release (Figure 1, Figure 2 and Figure S1) and COPII was colocalized with the PEDV N protein (Figure 3). To determine whether HDAC-specific inhibitors promote the colocalization of COPII and PEDV N protein, Vero-E6 cells were infected with PEDV with or without TSA or NaB treatment. Vero-E6 cells were stained with anti-COPII antibody to identify transport vesicles. The colocalization of COPII and PEDV N was analyzed with confocal microscopy. PCC values showed that the colocalization of COPII and PEDV N protein was enhanced by HDAC-specific inhibitors (Figure 4A,B). These findings indicated that the HDAC-specific inhibitors promote PEDV N proteins capture into the COPII-coated vesicles. Immunofluorescence showed that the HDAC-specific inhibitors reduced PEDV N protein levels (Figure S2).

### 3.4. Secretion of COPII-Coated Vesicles Carrying PEDV Virions Is Promoted by HDAC-Specific Inhibitors

**Figure 5:**
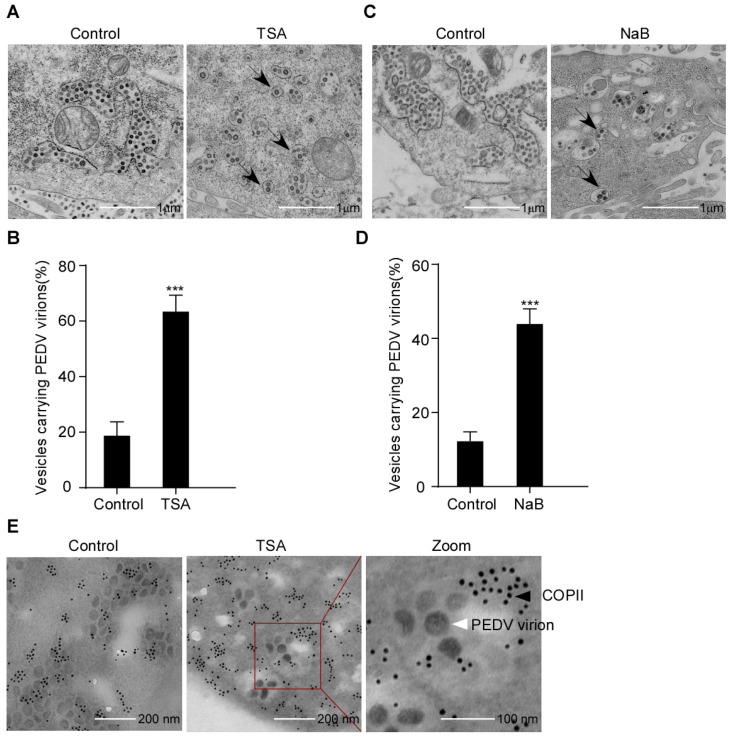
PEDV virions localized to COPII-coated vesicles following HDAC-specific inhibitors treatment. Vero-E6 cells were pretreated with or without TSA (60 ng/mL) and NaB (4 mM) for 2 h, and then mock infected or infected with HLJBY (MOI = 0.1). After HLJBY adsorption for 1 h, the cells were further cultured in fresh medium in the presence of TSA and NaB at 12 or 24 h. (**A**,**C**) Virus particles were detected with a transmission electron microscopy (TEM). Arrows indicate transport vesicles containing virions. (**B**,**D**) Graphs show the percentage of vesicles carrying PEDV virions. Student’s *t* test was used for statistical analysis. ***, *p* < 0.001. The error bars indicate standard deviation from three independent experiments. (**E**) Detection of COPII-coated vesicles by immunoelectron microscopy (IEM). The red box shows the PEDV virions enclosed in COPII-positive vesicles. The black arrow points to gold nanoparticles (10 nm)-labeled COPII. The white arrow points to PEDV virions.

To establish the PEDV secretion induced by HDAC-specific inhibitors, we analyzed the inhibitory effect on PEDV compartmentation using TEM. Vero-E6 cells were infected with PEDV and treated with or without TSA or NaB. The PEDV virus particles were detected by TEM. As expected, it was found that PEDV virions mainly accumulate in ER-like luminal structures in control cells. In contrast, PEDV virions in the HDAC-specific inhibitors-treated cells were mainly enclosed within transport vesicles; the arrows indicate that some transport vesicles containing only one virion and others containing multiple virions (Figure 5A–D). These findings suggested that the HDAC-specific inhibitors promote the secretion of transport vesicles containing PEDV virions. In order to verify whether the transport vesicles were COPII-coated vesicles, we performed IEM to analyze the COPII-coated vesicles for PEDV virions. PEDV was found to be enclosed within COPII-coated vesicles (Figure 5E). The data showed that the HDAC-specific inhibitors promote the secretion of COPII-coated vesicles carrying PEDV virions.

### 3.5. HDAC-Specific Inhibitor-Promoted Secretion of PEDV Is Dependent on COPII Complex

**Figure 6:**
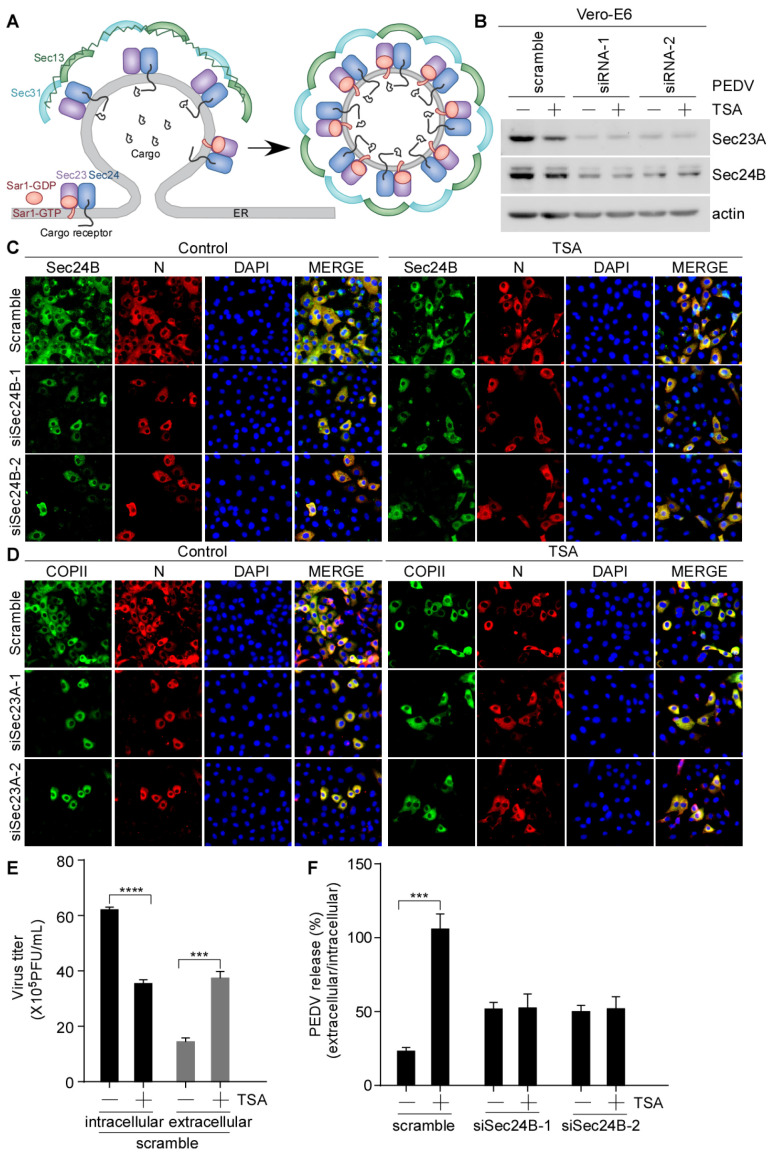
Suppression of HDAC-specific inhibitors-induced PEDV secretion by Sec24B knockdown. (**A**) Schematic diagram of the vesicle budding process in the COPII complex. Vero-E6 cells were transfected with nontargeting scrambled siRNA or targeting Sec23A/Sec24B siRNA for 48 h, and then pretreated with or without TSA (40 ng/mL) for 2 h, followed by HLJBY (MOI = 0.1) infection. After HLJBY adsorption for 1 h, the cells were further cultured in fresh medium in the presence of TSA for 12 h. (**B**) Sec23A/Sec24B and actin were detected by immunoblotting. (**C**,**D**) The cells were fixed and stained with Sec24B or COPII antibody and Alexa 488-conjugated goat anti-rabbit IgG antibodies (green) and then stained with PEDV-N antibody and Alexa 555-conjugated goat anti-mouse IgG antibody (red). The nuclei were stained with DAPI (blue). The images were acquired with a Nikon immunofluorescence microscopy. (**E**) Virus titers were measured via plaque formation assay, and the graphs show changes in virus titers (intracellular and extracellular); 12 h (×10^5^). (**F**) Graph shows PEDV release (ratio of extracellular to intracellular virus titers). Student’s *t* test was used for statistical analysis. ***, *p* < 0.001; ****, *p* < 0.0001. The error bars indicate standard deviation from three independent experiments.

The COPII complex consists of five subunits: Sar1, Sec23, Sec24, Sec13 and Sec31 [23]. Sec23 acts as a GAP for Sar1. Sec24 is a primary subunit of the COPII-coated vesicles, which is responsible for the transportation of cargos from the ER to the Golgi apparatus [23,45,46]. A schematic diagram of the COPII-coated vesicles formation is presented in Figure 6A. To further establish the correlation between the COPII-coated vesicles and virus release, we disrupted the formation of the COPII-coated vesicles by knocking down Sec23A or Sec24B. Vero-E6 cells were transfected with Sec23A or Sec24B small interfering RNA (siRNA), followed by infection with PEDV with or without TSA treatment. The endogenous levels of Sec23A and Sec24B were significantly decreased in cells treated with Sec23A/Sec24B-specific siRNA compared with cells transfected with nontargeting scrambled siRNA (Figure 6B). Immunofluorescence showed that TSA reduced the PEDV N level in the cells transfected with non-targeting siRNA. However, TSA treatment had no significant effect on PEDV N levels in cells treated with Sec23A or Sec24B-specific siRNA (Figure 6C,D). The PFU data showed that TSA decreased the intracellular virus titer and increased the extracellular virus titer in cells with non-targeting siRNA transfected cells (Figure 6E). PEDV release was evaluated using the ratio of extracellular-to-intracellular virus titers. We found that PEDV release by TSA was inhibited in cells with Sec24B knockdown cells (Figure 6F). The above results indicated that Sec24B-containing COPII-coated vesicles are essential for PEDV release following HDAC-specific inhibitors treatment. Interestingly, we found that the decrease in PEDV N protein levels induced by HDAC-specific inhibitors leads to a decrease in Sec23A/Sec24B protein levels (Figure S3).

## 4. Discussion

**Figure 7:**
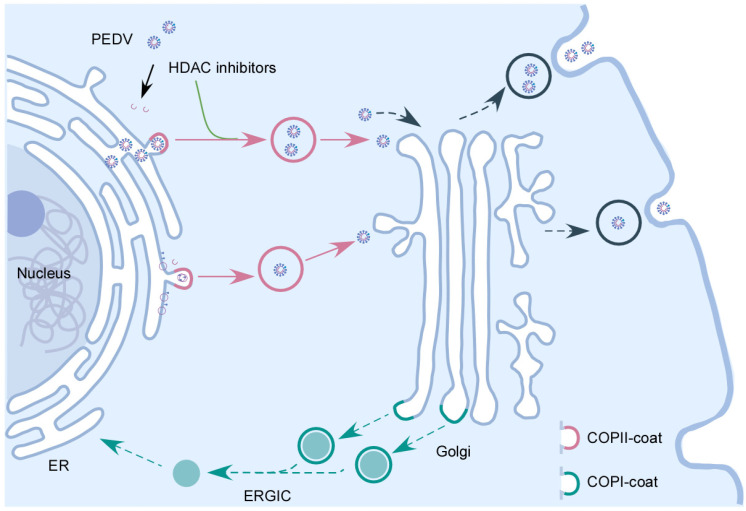
The interplay between HDAC-specific inhibitors and COPII-coated vesicles during PEDV infection. Following the entry of PEDV into host cells and replication in the cytoplasm, the newly synthesized PEDV viral components and PEDV virus particles are transported to Golgi apparatus by the COPII-coated vesicles. HDAC-specific inhibitors facilitate PEDV release by promoting the secretion of COPII-coated vesicles carrying PEDV virions.

PED is a highly contagious viral disease in pigs. PEDV infection results in high mortality in neonatal suckling piglets [47,48]. Similar to other enveloped RNA viruses, PEDVs uses and modifies intracellular compartments of the secretory pathway to facilitate viral replication and egression [13]. However, the underlying mechanisms of PEDV secretion remain largely unknown. In this study, we demonstrated that the HDAC-specific inhibitors hijack the COPII-coated vesicles to promote PEDV release (Figure 7). HDAC inhibitors have been reported to induce antiviral effects against a few enveloped RNA viruses, including RSV, HCV and SARS-CoV-2. TSA and SAHA can suppress RSV and HCV replication [38,39,40]. Romidepsin prevents the entry of SARS-CoV-2 [41]. In the present study, we found that TSA and NaB promote the secretion of PEDV virus particles into the extracellular compartment (Figure 1 and Figure S1). Consistently, the PFU data showed that the HDAC-specific inhibitors facilitate the secretion but not assembly of infectious PEDV particles (Figure 2).

Virion assembly occurs in the cytoplasmic side of the ERGIC/Golgi compartments in CoVs. Alpha-coronaviruses (TGEV, HCoV-NL36) and beta-coronaviruses (MHV) can assemble in the ER at later stages of virus infection [8,9,10,11,12]. PEDV has been reported to assemble in both the ER and Golgi apparatus, and the newly assembled virus particles subsequently utilize the secretory pathway for egression [13]. In our study, we observed the accumulation of PEDV virions mainly in the expanded ER lumen of control cells. In contrast, PEDV virions were mainly enclosed within transport vesicles in the HDAC-specific inhibitors-treated cells (Figure 5A–D). Further, we found that COPII-coated vesicles carried PEDV virions (Figure 5E). Thus, the results indicated that the HDAC-specific inhibitors promoted the secretion of COPII-coated vesicles carrying PEDV virions. HDAC-specific inhibitors promote the release of PEDV, and the virus could utilize the COPII-coated vesicles for intracellular trafficking. Vesicle transport requires a group of conserved proteins, such as Rab GTPases, motor adaptors and motor proteins to ensure vesicle transport along the cytoskeletal track [17]. HDAC inhibitors are able to affect cell transport, the promotion of IAV virion release by tubacin via acetylated microtubules [49,50]. Therefore, whether TSA and NaB promote the movement of the COPII-coated vesicles carrying PEDV virions along cytoskeletal remains to be determined.

COPII-coated vesicles promote the intracellular transport of cargos from the ER to the Golgi apparatus. COPII-coated vesicles have been reported to mediate in intracellular transport of several viruses, such as Ebola virus, Marburg virus, hepatitis B virus, HCV, parvovirus and rotavirus [30,31,32,33,34]. Here, we demonstrated that the HDAC-specific inhibitors-induced COPII-coated vesicles are essential for PEDV release (Figure 6). These findings also suggest that PEDV assembly is inhibited by Sec23A or Sec24B depletion (Figure 6). This indicated that the COPII-coated vesicles not only mediate the transport of PEDV virion assembled in the ER, but also mediate the transport of PEDV viral protein from the ER to Golgi for assembly. Our study is consistent with previous studies reporting that PEDV particles can be assembled in both the ER and Golgi apparatus [13].

It is generally assumed that virions that assemble in the ER are exported via the COPII-mediated early secretory pathway; the virions first reach the Golgi apparatus and TGN, followed by transport to the plasma membrane and egress [8]. However, a few studies reported that the beta-coronavirus MHV use lysosomes for egress and that the enteroviruses (poliovirus) and classical swine fever virus (CSFV) release occurred via autophagy pathway [10,51,52]. Whether PEDV viral particles in COPII-coated vesicles may use the biosynthetic secretory pathway or autophagosomes/lysosomes to egress requires further investigation. In summary, our findings reveal that PEDV virions assemble in the ER and bud off from the ER in the COPII-coated vesicles. We provide clear evidence to show that the HDAC-specific inhibitors hijack the COPII-coated vesicles to promote PEDV release.

## Data Availability

Not applicable.

## Supplementary Materials

The following supporting information can be downloaded at: https://www.mdpi.com/article/10.3390/v15091874/s1. Figure S1. HDAC-specific inhibitors promote the secretion of PEDV virus particles into the extracellular compartment. Vero-E6 cells were pretreated with or without TSA (40 ng/mL) and NaB (2 mM) for 2 h, and then mock-infected or infected with HLJBY (MOI = 0.1). After HLJBY adsorption for 1 h. The cells were further cultured in fresh medium in the presence of TSA and NaB at 6, 12, 18, 24 and 30 h. The infected cell lysates were prepared, and AC-H3, intracellular N and actin were detected by Western-Blot (A,C). The culture medium was divided into two parts; one part was analyzed by Western blotting of extracellular N (A,C), and the other part was titrated by plaque formation assay to measure the extracellular PEDV virus titers (B,D). Graphs show changes in virus titers (E,F). Student’s *t* test was used for statistical analysis. *, *p* < 0.05; **, *p* < 0.01; ***, *p* < 0.001. The error bars indicate standard deviation from three independent experiments. In; intracellular, Ex; extracellular; Figure S2. PEDV N levels are decreased by HDAC-specific inhibitors. Vero-E6 cells were pretreated with or without TSA (60 ng/mL) and NaB (4 mM) for 2 h, and then infected with HLJBY (MOI = 1) for 1 h. The cells were further cultured in fresh medium in the presence of TSA and NaB at 8 h. The cells were fixed and stained with COPII antibody and Alexa 488-conjugated goat anti-rabbit IgG antibodies (green) and then stained with PEDV-N an-tibody and Alexa 555-conjugated goat anti-mouse IgG antibody (red). The nuclei were stained with DAPI (blue). The images were acquired with a Nikon immunofluorescence microscopy; Figure S3. Sec23A and Sec24B levels are increased by PEDV infection. Vero-E6 cells were pretreated with or without TSA (40 ng/mL) and NaB (2 mM) for 2 h, and then infected with HLJBY (MOI = 0.1) for 1 h. The cells were further cultured in fresh medium in the presence of TSA and NaB at 12 h. The infected cell lysates were prepared, and AC-H3, Sec23A, Sec24B and actin were detected by Western-Blot.
