# Supplementary material for: HDAC-Specific Inhibitors Induce the Release of Porcine Epidemic Diarrhea Virus via the COPII-Coated Vesicles
Source: Viruses. 2023 Sep 4;15(9):1874. doi: 10.3390/v15091874 (PMC10534748; doi:10.3390/v15091874)
Supplement: Supplementary file 1 [file viruses-15-01874-s001.zip › viruses-2510565-supplementary-updated.pdf]

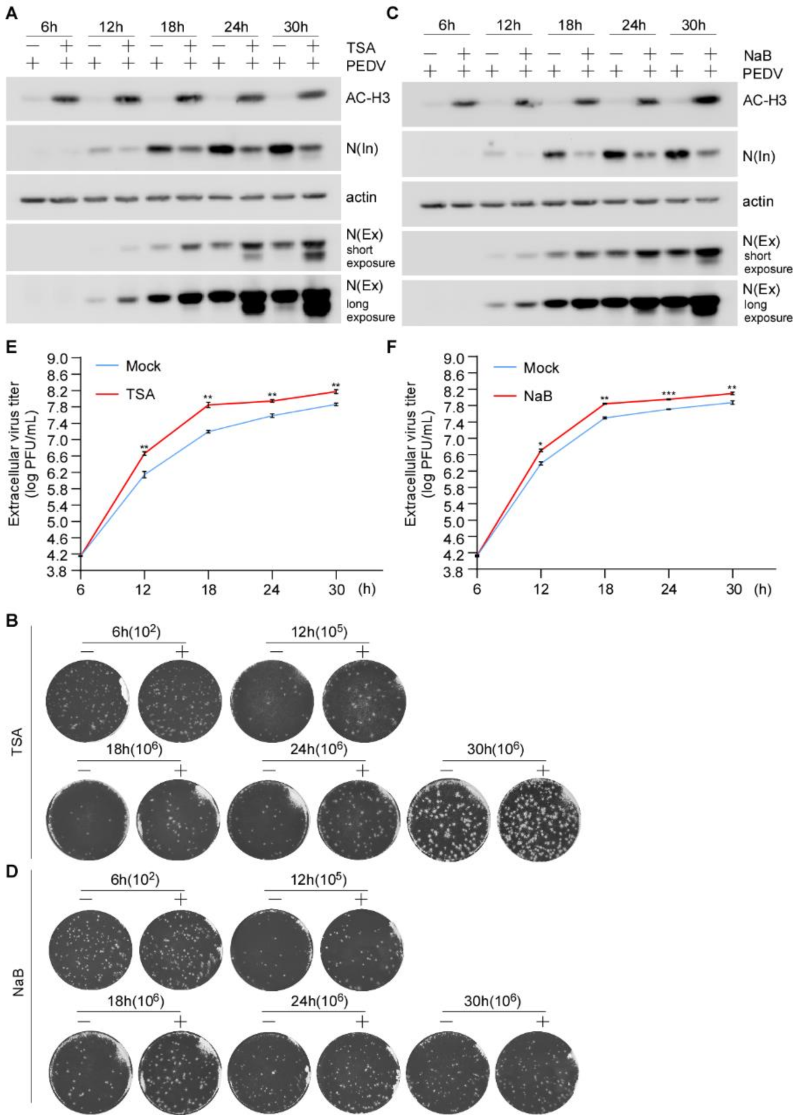

Figure S1. HDAC-specific inhibitors promote the secretion of PEDV virus particles into the extracellular compartment. Vero-E6 cells were pretreated with or without TSA (40 ng/mL) and NaB (2 mM) for 2 h, and then mock-infected or infected with HLJBY (MOI=0.1). After HLJBY adsorption for 1 h. The cells were further cultured in fresh medium in the presence of TSA and NaB at 6, 12, 18, 24 and 30 h. The infected cell lysates were prepared, and AC-H3, intracellular N, and actin were detected by Western-Blot (A,C). The culture medium was divided into two parts; one part was analyzed by Western blotting of extracellular N (A,C), and the other part was titrated by plaque formation assay to measure the extracellular PEDV virus titers (B,D). Graphs show changes in virus titers (E,F). Student's t test was used for statistical analysis. *, $P < 0.05$; **, $P < 0.01$; ***, $P < 0.001$. The error bars indicate standard deviation from three independent experiments. In; intracellular, Ex; extracellular.

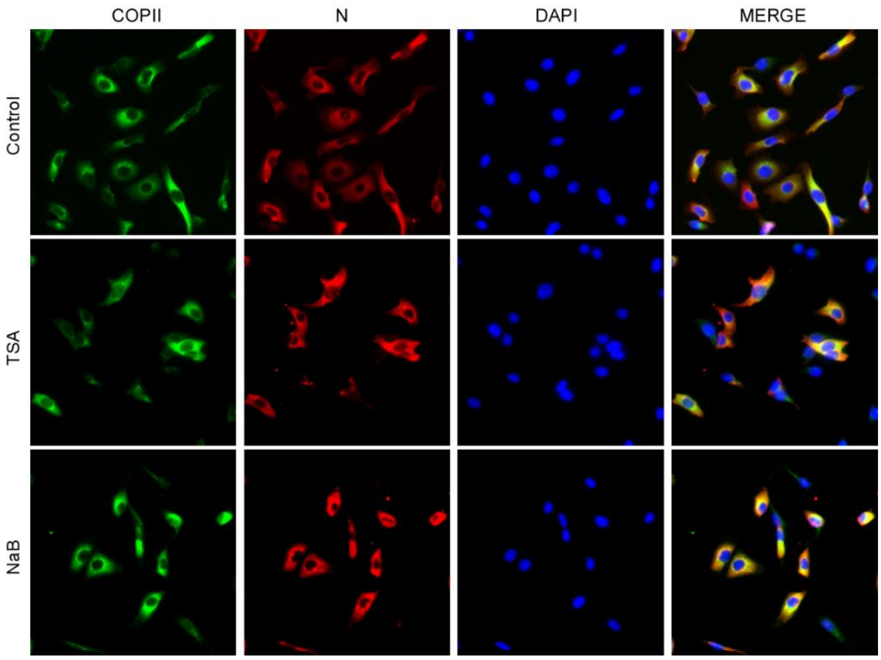

Figure S2. PEDV N levels are decreased by HDAC-specific inhibitors. Vero-E6 cells were pretreated with or without TSA (60 ng/mL) and NaB (4 mM) for 2 h, and then infected with HLJBY (MOI=1) for 1 h. The cells were further cultured in fresh medium in the presence of TSA and NaB at 8 h. The cells were fixed and stained with COPII antibody and Alexa 488-conjugated goat anti-rabbit IgG antibodies (green) and then stained with PEDV-N an-tibody and Alexa 555-conjugated goat anti-mouse IgG antibody (red). The nuclei were stained with DAPI (blue). The images were acquired with a Nikon immunofluorescence microscopy.

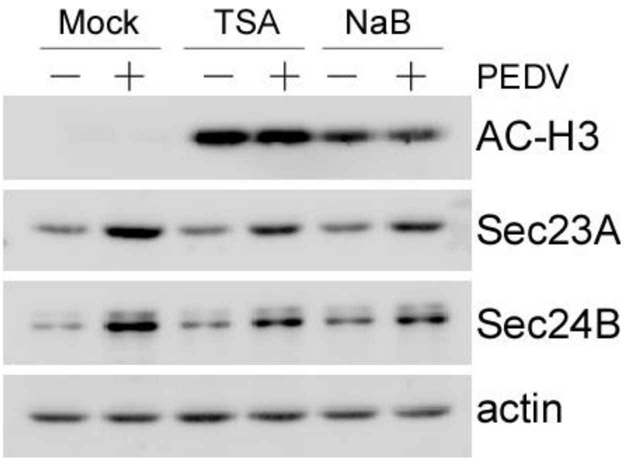

Figure S3. Sec23A and Sec24B levels are increased by PEDV infection. Vero-E6 cells were pretreated with or without TSA (40 ng/mL) and NaB (2 mM) for 2 h, and then infected with HLJBY (MOI=0.1) for 1 h. The cells were further cultured in fresh medium in the presence of TSA and NaB at 12 h. The infected cell lysates were prepared, and AC-H3, Sec23A, Sec24B and actin were detected by Western-Blot.
